# Correction: Recent updates in the discovery and development of novel antimalarial drug candidates

**DOI:** 10.1039/c8md90009d

**Published:** 2018-03-02

**Authors:** John Okombo, Kelly Chibale

**Affiliations:** a Department of Chemistry , University of Cape Town , Rondebosch 7701 , South Africa . Email: kelly.chibale@uct.ac.za; b South African Medical Research Council Drug Discovery and Development Research Unit , Department of Chemistry and Institute of Infectious Disease and Molecular Medicine , University of Cape Town , Rondebosch 7701 , South Africa

## Abstract

Correction for ‘Recent updates in the discovery and development of novel antimalarial drug candidates’ by John Okombo *et al.*, *Med. Chem. Commun.*, 2018, DOI: ; 10.1039/c7md00637c.



## 

The authors regret that the structures for compounds P65 (**44**) and P218 (**45**) in Fig. 5 were not correct in their manuscript. The corrected structures are shown below.
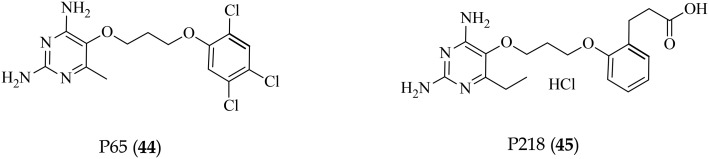


The Royal Society of Chemistry apologises for these errors and any consequent inconvenience to authors and readers.

